# Editorial Chit-Chat

**Published:** 1879-01

**Authors:** 


### Editorial Chit-Chat.

PREMIUMS.—Read our new premium list and observe the rewards we offer to those who work for the BISTOURY.

RELATIVES OF DISTINGUISHED MEN.—There are residing in this city a grand-daughter of Noah Webster, a nephew and several grand nephews of General Lee.

"ALWAYS AGAINST THE WOMAN."—Mr. Towner was unable to finish the second part of this article in season for the January number. It will appear in our next.

CITY SUBSCRIPTION LIST.—Thad S. Up de Graff, Jr., will receive and collect subscriptions for the city. We will recognize his receipt for moneys paid on account of subscription.

NOT EXEMPT.—Royalty, even, is not exempt from the scourge of diphtheria. Princess Alice, second daughter of Queen Victoria, died Dec. 14, of this disease, having previously lost a child from the same complaint.

THE ELMIRA PRISON.—Dr. Dudley, formerly surgeon of the 14th Regt. of Conn. Infantry, and who now resides in King's Ferry, N. Y., has written a letter to the BISTOURY, suggested by reading Sergt. Potter's complaints of the Elmira prison. Dr. Dudley makes no charges against the managers of Libby Prison, believing that they did the best they could for the northern prisoners there confined, but thinks their *best* food was so far inferior to our *worst*, that it would be well enough to call Sergt. Potter's attention to this fact.

LIFE INSURANCE COMPANIES.—The various life insurance companies of the country are becoming very much exercised over the success of Relief Associations. The latter are seriously interfering with the business of the former, so that presently it will be impossible for the presidents of the large life companies to draw their munificent salaries of $25,000 a year; or, perhaps, to erect the costly and imposing edifices that grace and ornament our cities. Unless the regular life companies reduce their prodigious expenses, and afford insurance at reasonable rates, they will surely disappear before the safe and steadily advancing relief associations that are furnishing insurance at from ten to twelve dollars on the thousand, and who *never* dispute a death claim, but pay promptly and cheerfully.

PARK CHURCH CHOIR.—The dime concerts given by this splendid choir are abundantly patronized, hundreds of people being unable to gain admission to the spacious church. Mr. Perry contemplates a concert every two weeks, hereafter, to satisfy the public demand for their excellent music.

AQUÆ GLORIA.—The quarterly journal bearing this name has suspended. We are sorry. Dr. Wales made a sprightly and exceedingly entertaining periodical of it. Indeed, the Doctor has all the "surface indications" of a first-class journalist. We hope he has not laid down his pen for any considerable time.

SUBSCRIPTION BLANK enclosed. Pay up promptly and send us a new subscriber if you can. Look over the table of contents, and see what an array of information is given you for fifty cents a year. No Journal in existence is so valuable to the family, and none more so to the physician. Our medical items and news are culled from the best medical journals in the land, to secure which, would cost more than fifty dollars a year. We offer *all* the news and hints they contain for fifty cents.

MARRIAGE OF OLD PEOPLE.—A conversation at a dinner-table recently brought out many comical stories of the wedding of old men to young wives. The desire for old men to re-wed was commented upon at length, when a gray-haired doctor, sitting to our right, convulsed the diners with laughter when he had remarked, in measured accents: "Well, I tell my wife to hang on—hang on to the bitter end, because if she don't, first thing you know, I'll be making a donkey of *my*self."

HOW MANY APPLES?—"Some say Eve 8 an apple and Adam 2; total, 10. Now we figure the thing differently. Eve 8 and Adam 8 also—total, 16."—*Boston Journal.*

"We think the above figures entirely wrong. Eve 8 1st and Adam 8-2—total, 163."—*Gloucester Advertiser.*

"Wrong again. What could be clearer than if Eve 8-1 and Adam 8-1-2, the total was 893?"—*Lawrence American.*

"But if Eve 8-1-1st and Adam 8-1-2, would not the whole be 1,623?"—*Boston Journal.*

Still another calculation is as follows: If Eve 8-1-4 Adam, Adam 8-1-2-4-2 oblige Eve; total, 82,056.—*New York Mail.*

Yes, but if Adam 18-1-2,4-2 keep Eve company as claimed, and Eve 8-1-2-4 Adam, it is plain that 189,366 apples were consumed in the transaction.

THAT HISTORY.—A few of our citizens were inveigled into patronizing what purports to be a history of Tioga, Chemung, Tompkins and Schuyler counties, in this State. The historical part is perhaps well enough, but the picture gallery—Oh, the portraits! :—well, they're funny!

Had the publishers permitted portraits only of our oldest and most distinguished citizens, and excluded all the rest, they would have made a presentable book; but when every fellow's portrait is given who signifies a willingness to pay $55 for the privilege of seeing himself displayed in the "History," why, the thing becomes grotesque.

---

ECONOMY, when well directed, is appreciated of all men. Sometimes it happens that an effort at economy fails of its intended result. Indeed, there are times when it proves disastrous. As witness a case in point:

Our Board of Supervisors have been industriously reducing the annual bills of the Sheriff and other parties; have curtailed the salaries of certain county employees—excepting their own, so *saving* to the county several thousand dollars, as they claim. But, in accomplishing this result, they have doubled the days of their sitting, (promptly drawing their salaries the while) have entangled themselves in expensive legal controversics, have employed the best council—necessarily expensive —by all of which procedures, they have incurred an expense to the tax-payers of the county exceeding the sum sought to be saved.

There would seem to be too much politics and too little common sense in that Board of Supervisors.

---

THE PRISON CAMP.—The *Daily Advertiser*, of this city, recently published an article from a Sergt. Potter, of Tennessee, in which he made sundry statements relative to the food and management of the prison camp in this city during the war. The Sergeant charged cruelty upon the confederate prisoners by the prison officers, complained of a scarcity of food, clothing and fuel, with a vindictiveness and recklessness for the truth by no means of credit to him. We are glad to see a denial of his statements, *in toto*, from so high an authority as General Diven. As the BISTOURY has a large circulation in the Southern States, we desire to assure our readers in that section that Mr. Potter's assertions are without an atom of truth. We frequently visited the quarters of the prisoners, and can testify that they were more comfortable and healthful than those of our own troops, while the food was wholesome and abundant. If any cruelty was practised upon the prisoners, it certainly was never known here, nor ever complained of by the inmates of the prison camp.

Sergeant Potter will reap neither fame nor credit from his ungenerous and libelous letter.

---

A PLUCKY LITTLE WOMAN.—Sarah A. Hibbard, M. D., of Rochester, N. Y., was recently prosecuted by a Mr. Hibbard, of the same city, for alleged malpractice in setting a broken arm. The prosecutor offered to settle with the Doctor for a few hundred dollars, but she preferred to fight it out, claiming that she did as good a job as any surgeon could have accomplished in any similar fracture. The case came to trial, and, notwithstanding the male doctors tried to have the case go against her, she came off victorious, was acquitted, and the costs of prosecution assessed to the man bringing the suit. Served him right. Nine-tenths of the suits for malpractice in such cases are brought with a view of blackmailing the surgeon. We are glad the little woman stood her ground, setting an example to surgeons of the sterner sex.

---

LAUGHING GAS.—Fred S., suffering severely from toothache, after long persuasion from father and mother to have the offending tooth extracted, finally consented after a proffered reward of a silver dollar. With his father, he repaired to the dentist's office, where gas was to be administered that the operation might be a painless one. Fred, seated in the great chair, watched every movement of the dental man and plied him with sundry questions about the gas. Finally, the dentist appeared from an adjoining room, with an immense rubber bag, filled with the nitrous oxide gas. Fred beheld it with manifest symptoms of alarm, and, with his great, blue eyes open to their widest extent, which he kept fixed upon the inflated bag, slid from the chair and backed toward the door.

"Here, here, where are you going? Come, you must take the gas now!" exclaimed the father.

"Goodness, I can never swallow that big thing!" said Fred, as he darted from the door and escaped to his home.

---

THE COOKING CLUB.—Such an institution exists in our city, composed of ladies and gentlemen, who congregate at the dwelling of one of their number once in two weeks, to partake of a supper to which every member contributes something of their own cooking. The gentlemen persist in furnishing such easily cooked (?) dishes as raw oysters, celery, grapes, cut cabbage, and the like, while the ladies astonish each other with more complicated products of the culinary art.

At a recent sitting of the club, a lady brought to the table a pudding, over which brandy had been poured and ignited, sending a blaze almost to the ceiling. The effect was somewhat startling, and suggested an idea to one of the gentlemen members which he placed in execution at the very next meeting.

He prepared an enormous Indian meal pudding, and, when the guests were all seated about the table, carried it into the room, with a sky-rocket spitting fire in its centre, and a bunch of exploding fire-crackers tied to each handle of the pudding dish. His exploit cleared the table and the room, and there has been no meeting of the club since.

---

FRITZ AT SCHOOL.—Fritz was offered by his mother, as an incentive to study, a dime if he attained the head of his class at school. A visitor in the family promised another dime, while the father agreed to double the sum in aid of the project. Fritz was delighted at the prospect of earning forty cents, particularly as the holidays were close at hand with their attractions for money-spending, and his black eyes danced and sparkled with pleasure and mischief.

"I'll win yer forty cents or bust," was his joyful exclamation as he skipped out of the dining-room, bounded over the back fence into the lane, running like a quarter horse to reach the school before the bell should cease ringing.

It must be known that Fritz was at the foot of his class, where he persisted in remaining, assigning as a reason that he had a "soft thing of it there, and didn't need to study like them other fellers to keep up head all the time."

Upon the following day, at the tea-table, Fritz was interviewed as to his progress.

"Well, I've scooped fourteen of 'em, an' if them other twenty-four fellers 'll only let up a little, an' not study so pleagy hard, I'll fix 'em too."

A week passed by, when Fritz joyfully announced that he was within three of the head. At the end of another week, he had not gained upon those three, and his interest in school and monetary affairs seemed to flag, requiring much coaxing and encouragement upon the part of his mother to have him retain his position in the class.

At last, a bright idea struck him, worthy even of Tom Sawyer. And this was the conversation we overheard with his brother, under our sanctum window:

"I say, Way."

"Well, what?"

"I know what I'll do, by golly. I just thought of it! I'll give Tim—he's head, you know—my old top; and Pat—he's next—a cent's worth of peanuts, 'cause he's awful fond of 'em —; and I'll hook an apple from grandma's cellar for George, if they'll all miss to-day and let me go up head. Let's see—then I'll have thirty-nine cents left! Say, Way, havn't you sumthin, I can give Pat, so's not to buy the peanuts, 'cause then I can save the whole pile of money, you know?"

The school bell rang;—both boys departed. In the evening, Fritz was seen approaching his home on a keen run. He rushed into his mother's presence, shouting and throwing up his hat. Then, through the telephone, we heard this announcement:

"Fritz has reached the head of his class!"

---

STEWART'S BODY.—H. has repeatedly said in our hearing that, "it is getting dangerous for a man to try to live." He might have added, with equal clearness, "and more so to die," particularly for those who have any solicitude as to the disposition of their bodies. There seems to be no security or certainty for the repose of a man's body, unless he sits upon his tombstone and guards his own grave with a Winchester repeating rifle. We do not believe that Stewart cares a cent where his body is, but many excellent people do, and now that body-snatchers are beginning to speculate upon rich corpses, either a fellow must die poor or lock himself up in a burglar proof safe before he becomes a cold corpus.

The rational way to prevent such traffic is to have your body cremated. It is a sensible way, too, better for the living and the dead.

The editor of the Saturday Miscellany says "if there be ten men in this community, of the same mind with us, there will be a cremation furnace, with a little chapel around it, built within six months."

Put us down for one of the ten.

By-the-way, why could not one of the retorts at the gas-works be made to do duty in cremating a body? They can be sufficiently heated, and, if we remember rightly, are large enough to receive an adult body. If this be so, then have we a capital cremating furnace ready for occupancy. When Edison's electric light is perfected and gone into use, the gas companies can find a new and profitable business by converting their ovens into cremating furnaces. Why not?

